# Acute liver failure due to radiographically occult infiltration of urothelial cancer

**DOI:** 10.4322/acr.2021.256

**Published:** 2021-03-12

**Authors:** Valentina Tosatto, João Cabral Pimentel, Cristiano Cruz, André Almeida, Matteo Boattini

**Affiliations:** 1 Centro Hospitalar Universitário de Lisboa Central, Hospital de Santa Marta, Department of Internal Medicine 4, Lisboa, Portugal; 2 Universidade Nova de Lisboa, NOVA Medical School, Lisboa, Portugal.; 3 Centro Hospitalar Universitário de Lisboa Central, Hospital de São José, Department of Anatomical Pathology, Lisboa, Portugal.; 4 University Hospital Città della Salute e della Scienza di Torino, Microbiology and Virology Unit, Turin, Italy.

**Keywords:** Neoplasms, Biopsy, Palliative Care

## Abstract

**Introduction:**

Acute liver failure (ALF) due to diffuse infiltrating solid malignancy without any focal lesions on radiographic imaging is rare.

**Case report:**

A 70-year-old man was admitted due to mental confusion, abdominal pain, and ALF. Three years before, he had undergone a left nephrectomy for urothelial carcinoma followed by adjuvant chemotherapy. The abdominal computed tomography (CT) showed hepatomegaly and ascites. Ascitic fluid had transudate characteristics, with no malignant cells. Percutaneous liver biopsy (LB) showed diffuse liver infiltration of metastatic urothelial carcinoma. The patient rapidly deteriorated and died in a week due to ALF.

**Discussion:**

History of solid cancer and hepatomegaly and/or liver failure without other obvious explanation should encourage to perform LB.

**Conclusion:**

LB is warranted to avoid misdiagnosis, prolonged hospital stays, and delay in palliative care.

## INTRODUCTION

Acute liver failure (ALF) is a life-threatening critical illness that occurs most often in patients with no previous liver disease history.^1^ Solid cancers commonly present with a primary lesion and metastasis to one or more organs easily detected by imaging, including computed tomography (CT) and magnetic resonance imaging (MRI). However, despite being rare, ALF due to infiltrating malignancy without any focal lesions on imaging can occur and is associated with high mortality. Neoplasms, including the gastrointestinal tract, breast, urothelial, lung, and nasopharynx cancers, as well as lymphomas, leukemias, and malignant histiocytosis, are the most involved.^2^^,^^3^ ALF results from diffuse sinusoidal infiltration, portal vein thrombosis, hepatic ischemia, and necrosis.^2^ In this report, we describe a case of a patient with ALF due to infiltrating urothelial malignancy. We highlight the importance of a high level of suspicion in preventing clinical hesitation to liver biopsy (LB), misdiagnosis, and delay in palliative care.

## CASE REPORT

**Figure 1:**
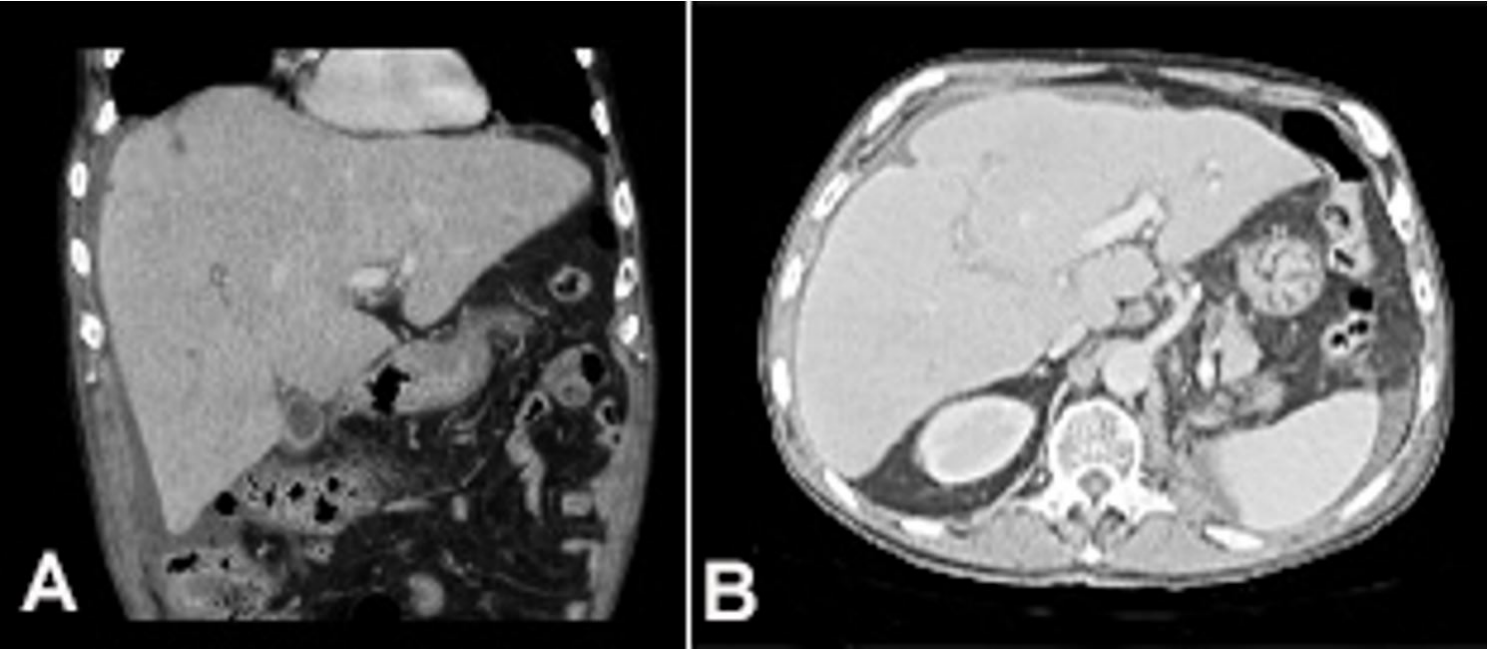
A and B - High-resolution abdominal CT showing hepatomegaly with diffuse inhomogeneous parenchyma, extensive periaortic lymph nodes involvement, and ascites.

**Figure 2:**
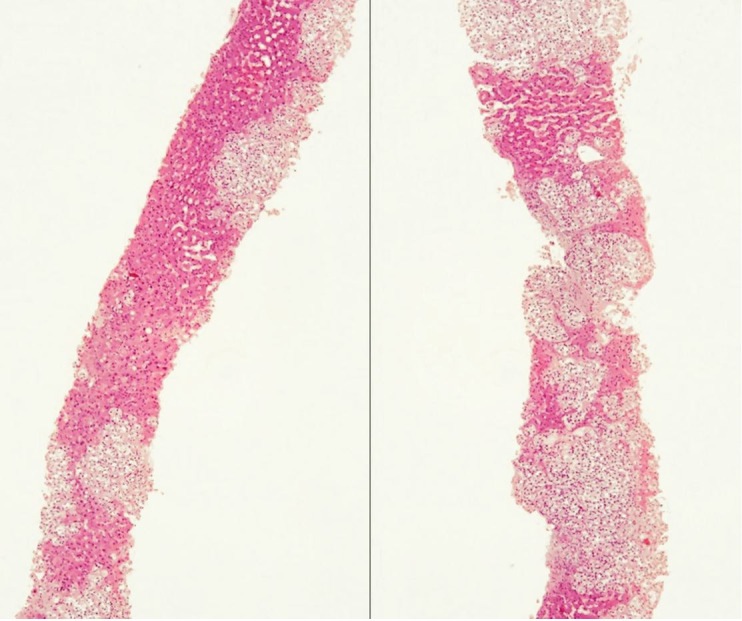
Photomicrograph of the liver biopsy showing diffuse liver infiltration by metastatic urothelial carcinoma. (H&E; 40x).

**Figure 3:**
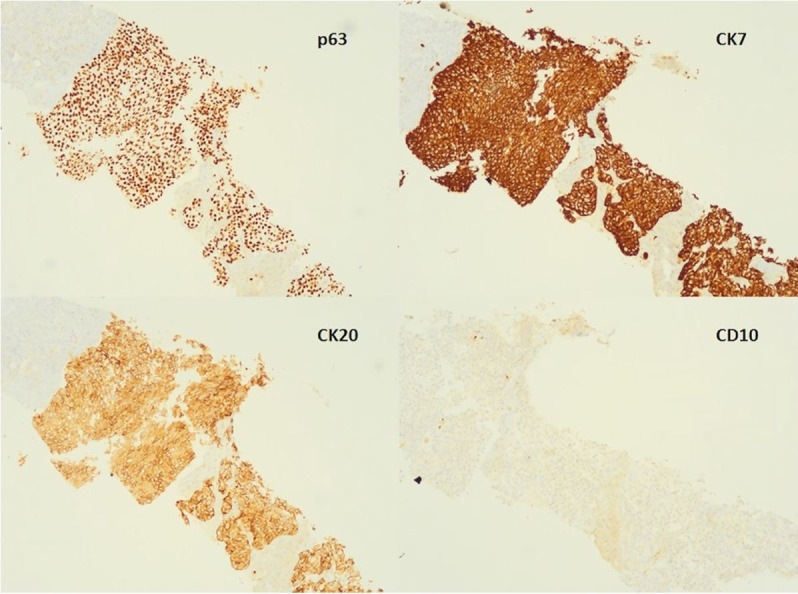
Photomicrograph of the liver biopsy showing positive reaction for p63, CK7, CK20 and negative reaction for CD10 in the malignant cells. (40x).

A 70-year-old man was admitted with a 3-day history of mental confusion, diffuse abdominal pain, nausea, and lethargy, which gradually worsened. No new medication was introduced in the previous days. Neither alcohol nor illicit drug consumption was reported. His medical history included a nephrectomy, followed by adjuvant chemotherapy (gemcitabine and carboplatin) to treat urothelial carcinoma, three years ago. On the physical examination, the patient was icteric and drowsy, with mild alteration of the sensorium. He had asterixis and grade II encephalopathy. His body temperature was 36 °C, pulse rate was 88 bpm, respiratory rate was 22/min, and blood pressure was 108/55 mmHg. Lung and heart examination was unremarkable, and moderate hepatomegaly, and ascites were detected on abdominal palpation. Blood examination showed in Table 1 was consistent with liver failure. Serology tests for viral hepatitis and HIV were negative, and antinuclear and anti-smooth muscle antibodies were undetectable. Head CT showed no intracranial space-occupying lesions. High-resolution abdominal CT showed hepatomegaly with diffuse inhomogeneous parenchyma, extensive periaortic lymph nodes involvement, ascites, with no genitourinary system lesions or peritoneal implants (Figure 1). Abdominal paracentesis was performed, draining an ascitic fluid with transudate characteristics and negative results for bacterial culture neither malignant cells. The patient underwent percutaneous LB showing diffuse liver infiltration by metastatic carcinoma (Figure 2) with immunohistochemical profile positive for p63, CK20, CK7, and negative for CD10 (Figure 3), suggesting urothelial origin. No complications related to LB occurred. Palliative care was promptly and properly initiated. Besides this, the patient rapidly deteriorated and died in a week due to the onset of ALF.

**Table 1:** Laboratory work up

Exam	Result	RR	Exam	Result	RR
Hemoglobin	10.7	11.5–15.5 g/dL	ALT	1154	<35 U/L
Leukocytes	7800	4500–11000 /µL	Gamma-GT	204	<38 U/L
Platelet	177	150–450 × 10^3^/µL	AP	192	30–120 U/L
Urea	39	17–43 mg/dL	LDH	568	<247 U/L
Creatinine	0.72	0.51–0.95 mg/dL	INR	1.98	0.8-1.2
TB	8.4	0.3-1.0 mg/dL	Ferritin	407	24–336 ng/mL
DB	5.6	0.1-0.3 mg/dL	Ceruloplasmin	35	14-40 mg/dL
AST	3190	<35 U/L	24-h urine copper	22	20-50 μg

ALT = alanine transaminase; AP= alkaline phosphatase; AST= aspartate transaminase; DB= direct bilirubin; Gamma GT= gamma glutamyl transferase; INR= international normalized ratio; LDH= lactate dehydrogenase, RR=reference range; TB= total bilirubin.

## DISCUSSION

Solid cancers mainly involved in the hepatic infiltrative metastatic pattern are lung, breast, renal and urothelial cancers, whereas there are anecdotal cases involving prostate, melanoma and neuroblastoma. Infiltrative liver disease caused by solid cancer metastases is a rare cause of ALF^4^ and usually presents with unspecific findings such as hepatomegaly, transudative ascites with the absence of malignant cells, high levels of aminotransferases, conjugated bilirubin and LDH. Despite widely reported,^5^^-^^9^ it is frequently diagnosed post-mortem as the first case due to diffuse metastases of urothelial carcinoma reported in 1996.^5^ Conventional imaging including both CT and MRI have been reported to show low performance in detecting metastatic infiltrative liver disease, probably due to the micro-invasion histological pattern.^7^^,^^10^ In cases with unspecific clinical, laboratory and imaging findings, the percutaneous/trans jugular LB, is the most accurate tool to obtain a proper diagnosis in patients with ALF of unknown cause. Although the risk of bleeding due to coagulation abnormalities and low platelet count should always be considered, LB seems to be one of the best prognostic tools in internal medicine, especially in older patients without a diagnosis, who would not be candidates for transplantation. Finally, given that ALF due to malignant infiltration of the liver could be seen both in patients with or without a history of solid cancer and given the poor prognosis, LB may expedite palliative care.

## CONCLUSION

History of solid cancer and hepatomegaly and/or liver failure without other obvious explanations should encourage to perform LB. LB is warranted to avoid misdiagnosis, prolonged hospital stays and delay in palliative care. The main key-points highlighted by this case are the following: (1) radiographically occult liver infiltration caused by solid metastatic cancer should always be considered as a cause of ALF; (2) conventional imaging examinations poorly detect diffuse metastatic infiltrative disease; (3) the LB may shorten the delay of the diagnosis and palliative care.
